# A Study on Railway Surface Defects Detection Based on Machine Vision

**DOI:** 10.3390/e23111437

**Published:** 2021-10-30

**Authors:** Tangbo Bai, Jialin Gao, Jianwei Yang, Dechen Yao

**Affiliations:** 1 School of Mechanical-Electronic and Vehicle Engineering, Beijing University of Civil Engineering and Architecture, Beijing 100044, China; 2108230420003@stu.bucea.edu.cn (J.G.); yangjianwei@bucea.edu.cn (J.Y.); yaodechen@bucea.edu.cn (D.Y.); 2 Beijing Key Laboratory of Performance Guarantee on Urban Rail Transit Vehicles, Beijing University of Civil Engineering and Architecture, Beijing 100044, China

**Keywords:** deep learning, rail surface defect detection, machine vision, YOLOv4, MobileNetV3

## Abstract

The detection of rail surface defects is an important tool to ensure the safe operation of rail transit. Due to the complex diversity of track surface defect features and the small size of the defect area, it is difficult to obtain satisfying detection results by traditional machine vision methods. The existing deep learning-based methods have the problems of large model sizes, excessive parameters, low accuracy and slow speed. Therefore, this paper proposes a new method based on an improved YOLOv4 (You Only Look Once, YOLO) for railway surface defect detection. In this method, MobileNetv3 is used as the backbone network of YOLOv4 to extract image features, and at the same time, deep separable convolution is applied on the PANet layer in YOLOv4, which realizes the lightweight network and real-time detection of the railway surface. The test results show that, compared with YOLOv4, the study can reduce the amount of the parameters by 78.04%, speed up the detection by 10.36 frames per second and decrease the model volume by 78%. Compared with other methods, the proposed method can achieve a higher detection accuracy, making it suitable for the fast and accurate detection of railway surface defects.

## 1. Introduction

With the prosperous development of the railway industry, the mileage, speed and density of operations continue to increase, and the inspection requirements for railways are further improved [1]. When it runs at high speed, the phenomena such as friction, rolling contact and elastic deformation occur between the train and the track surface. With the running time increasing, it will result in rail surface defects, such as rail wear, broken, peeling and cracks, which seriously threaten the safety of the rail transit system [2]. Therefore, it is particularly important to study the detection methods for railway surface defects.

As a traditional method for rail surface detection, manual inspection [3] is characteristic of time-consuming, labor-intensive [4] and low detection efficiency [5]. With the development of defect detection technology, many rail surface defect detection methods have emerged, such as ultrasonic flaw detection [6], eddy current flaw detection [7], three-dimensional detection [8], radar detection [9] and so on. The above methods are very effective in detecting internal defects. However, the signals generated by the defects on railway surfaces are very weak, and they are difficult to detect by the above methods. At the same time, the defect signals are easily interfered with by the surrounding environment, leading it difficult to achieve satisfying results. There is still a big margin for improvement in the detection technology of rail surface defects.

With the development of computer technology, the machine vision [10] method is applied to rail surface defect detection. Rail surface detection images are obtained by linear array cameras, and the images are automatically synthesized according to the required length. Defect data are obtained by manual screening from actual detection images for model training and testing. This method requires an analysis of rail surface defect information, gray information [11] and background information [12]. It needs to use a feature extraction algorithm [13] or to use an operator template and model-based threshold segmentation method [14] to detect rail surface defects. However, these methods are susceptible to defect characteristics that may lead to blind spot detection [15]. This makes it difficult for machine vision methods to obtain good detection performances.

In recent years, with the development of target detection technology and the neural network [16], deep learning frameworks have been proposed for the detection of various railway components. Liu et al. [17] proposed a method based on image fusion features and Bayesian compression image classification and recognition, which detected the status of fasteners by extracting improved edge orientation histograms (IEOH) and macroscopic local binary pattern (MSLBP) features. Cui et al. [18] segmented the fastener image into different parts to avoid the interference of the fastener fragments and tested the segmentation model in a real-time deep learning module.

In the application of a deep learning framework for rail surface defect detection, Xu et al. [19] proposed to improve the Faster R-CNN (Convolutional Neural Networks) for railway subgrade defect recognition. The improved method can obtain good performance, but it has disadvantages such as a slow detection speed and large detection model. Lu et al. [20] proposed to apply the combined U-Net graph segmentation network and damage location method for damage detection of high-speed railways. This method can obtain a high detection accuracy but has the limitations of slow detection speed and large model volume. Yuan et al. [21] proposed the application of MobileNetV2 to detect rail surface defects, which achieved high-speed real-time detection, but the detection accuracy was low. Faghih-Roohi et al. [22] proposed improved deep convolutional neural networks (DCNN) to efficiently extract and recognize image features, and a small batch gradient descent method was used to optimize the network for the automatic detection of track surface defects. This method requires a long time for network training. Song et al. [23] proposed a deep learning method where the YOLOv3 (You Only Look Once, YOLO) algorithm was used to detect rail surface defects. This method has a fast detection speed but low detection accuracy.

In order to solve the above problems, this paper proposes an improved YOLOv4 [24] rail surface detection method. It studies the use of the MobileNetV3 lightweight network as the backbone of YOLOv4. Depthwise separable convolution is applied for the PANet layer in YOLOv4 to further reduce the amounts of the parameters. It treats rail surface defect detection as an end-to-end regression problem and ensures the effectiveness of rail surface defect detection with a simplified network, improving the detection speed and accuracy. It provides a new idea for rail surface defect detection technology.

The main contributions of this paper are as follows: (1) The MobileNetV3 network is proposed to optimize the YOLOv4 model for rail surface defect detection, using depthwise separable convolution for the PANet layer in YOLOv4. This method optimizes the parameter quantity and model size and improves the detection speed. (2) Field tests are conducted on the track to collect data, a dataset is created with Gaussian noise added, and finally, a rail surface defect detection model is established. The test results show that the method used in the study can effectively detect rail surface defects.

The rest of this article is organized as follows. The second part discusses the theoretical background of YOLOv4 and depth separable convolution. The third part gives the technical route of the proposed method. The fourth part verifies the effectiveness of the method through practical application. Finally, the conclusion is drawn in Section 5.

## 2. Theoretical Background

**Figure 1:**
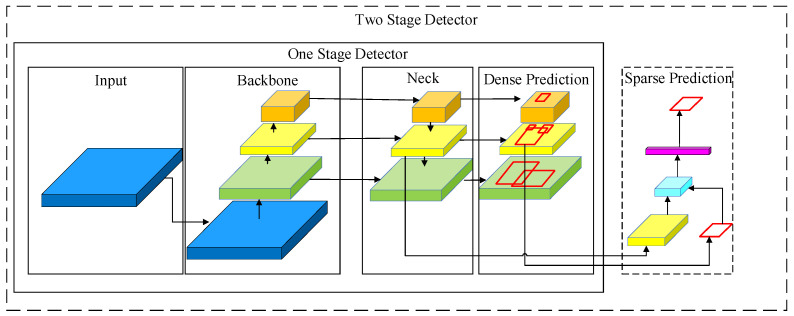
Object detector framework.

The deep learning and machine vision-based object detection methods are widely used in the current research. For the application of these methods, firstly, a large number of images is collected to establish the image datasets, and secondly, image annotation is performed on the object to be detected in the dataset to obtain the object information; then, a training dataset and the object information are trained by the deep network to obtain a deep network model, and finally, the trained model is used for the object detection test. Among them, the most important part is the training of the deep network model. At this stage, the target detector is mainly composed of four parts: input, backbone, neck and head. As shown in Figure 1, the structure of the one-stage network is simpler than the two-stage one, in which a sparse prediction is added.

Before the YOLO [25] algorithm was proposed, the R-CNN [26] algorithm was one of the most popular algorithms in the two-stage field. CNN has been applied to target detection and formed a relationship with R-CNN [27], the algorithm region. First, the selective search [28] or edge box of the algorithm is used to generate candidate regions [29], and then, each region is trained and classified in the CNN. Compared with the one-stage algorithms, the detection speed of the two-stage ones is slower. Therefore, a YOLO algorithm with the characteristics of the one-stage network structure is proposed. Its core concept is to convert the target detection into a regression problem, and the target map is used as the input of the network. Only through a neural network can the position of the bounding box and the target category be obtained. A fast detection speed and high precision can be realized through the feature information.

The YOLOv4 algorithm is improved from the basis of YOLOv3. As a powerful target detection algorithm, a fast and accurate target detector can be trained by YOLOv4. As shown in Figure 1, the network structure is mainly composed of a backbone network, a neck network and a head network. CSPDarknet53 is applied in the backbone network, an SPP add-on module and PANet path aggregation is performed in the neck network and the YOLOv3 head network is used as the head network.

**Figure 2:**
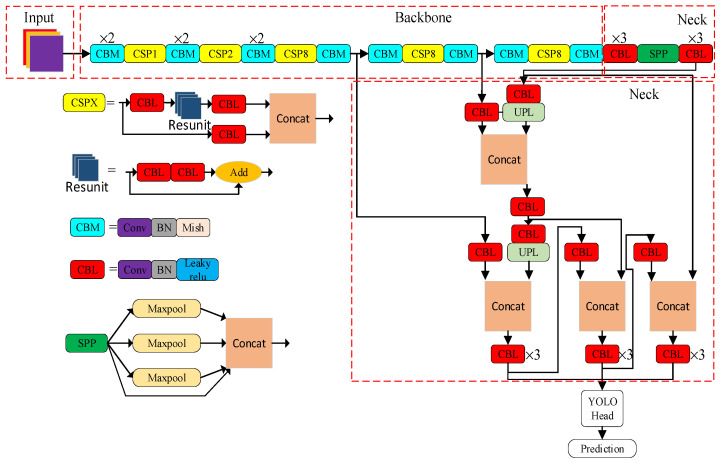
YOLOv4 structure diagram.

The PANet layer uses an instance segmentation algorithm. The network structure is shown in the neck part of Figure 2. Compared with the feature pyramid networks (FPN) network, the DownSample operation is added in PANet after UpSample to repeatedly improve the features. Parameter aggregation is carried out on the different backbone layers. It further improves the ability of feature extraction. In YOLOv4, the PANet structure is mainly used in the three effective feature layers.

## 3. Proposed Method

### 3.1. Technical Route

**Figure 3:**
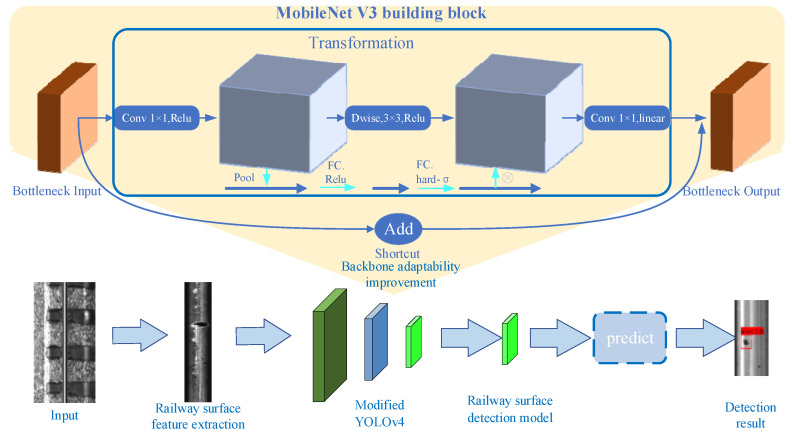
Technical route of the proposed method.

Figure 3 shows the technical route of rail surface defect detection. Firstly, feature extraction is performed on the whole rail image. While retaining the rail surface information, the invalid information is removed from the rail image to increase the network training speed. Secondly, the processed rail surface dataset is input into the improved YOLOv4 network for training. Then, the trained model is used to predict the rail surface defects. Finally, the rail surface defect detection results are obtained.

### 3.2. YOLOv4 Backbone Network Adaptability Improvement

In practical engineering applications, the detection of rail surface defects has particularities, including the accuracy, the speed and the model size of detection. The method in this paper takes into account the particularity of rail surface defect detection, making it adaptable to YOLOv4. MobileNetV3 is used as the backbone network of YOLOv4. MobileNet is a lightweight deep neural network proposed by Google for embedded devices. The core idea is the depthwise separable convolution. Compared with the traditional convolution used in YOLOv4, the deep separable convolution in MobileNetV3 can further reduce the amount of parameters and calculations, thus realizing the lightweight of the network.

**Figure 4:**
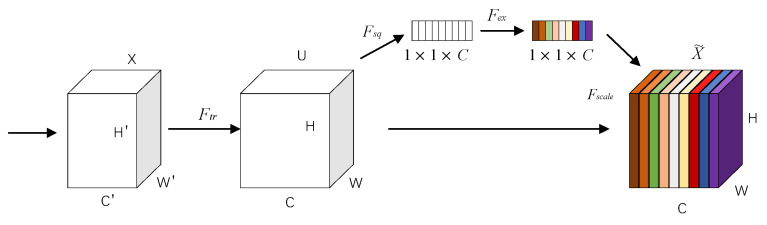
The RPN candidate box generation process.

A lightweight attention (Squeeze-and-Excitation, SE) module is used in MobileNetV3. Its advantage is that it can improve the performance of the algorithm with a negligible increase in the calculations. The specific process of the SE module is implemented as shown in Figure 4. First, the features of $C^{\prime }\times H^{\prime }\times W^{\prime }$ are optimized to C×H×W. Then, in the process of squeeze, global average pooling is performed on the C×H×W features to obtain a global receptive field feature map of 1×1×C in size. Then, a fully connected neural network is used for nonlinear transformation in the process of excitation. Finally, the input feature is weighted by the activation value of each feature layer from the SE module.

### 3.3. Adaptability Improvement of PANet Layer in YOLOv4

PANet in YOLOv4 has the advantages of dynamic feature pooling, fully connected layer fusion and bottom-up path enhancement but disadvantages such as a large amount of parameters and complex calculations. To resolve this problem, the convolution structure in PANet is modified, where the 3 × 3 and 5 × 5 standard convolutions are replaced by depth separable convolutions.

Depth separable convolution [30] is a lightweight convolution module. It consists of the following two parts: depthwise convolution (DW) and pointwise convolution (PW). In DW, each dimension in the input information is convolved with a convolution block separately. Then, PW applies a point convolution kernel to perform dimensional lifting of the output maps from DW.

In the standard convolutional layer, assume that the size of the input feature map is $D_{z}\times D_{z}$, the number of channels is M, the size of the convolution kernel is $D_{i}\times D_{i}$ and the number of convolution kernels is K. Then, the standard convolution calculation amount $C_{1}$ can be calculated by Formula (1):

(1) $$C_{1}=D_{z}\times D_{z}\times M\times K\times D_{i}\times D_{i}$$

**Figure 5:**
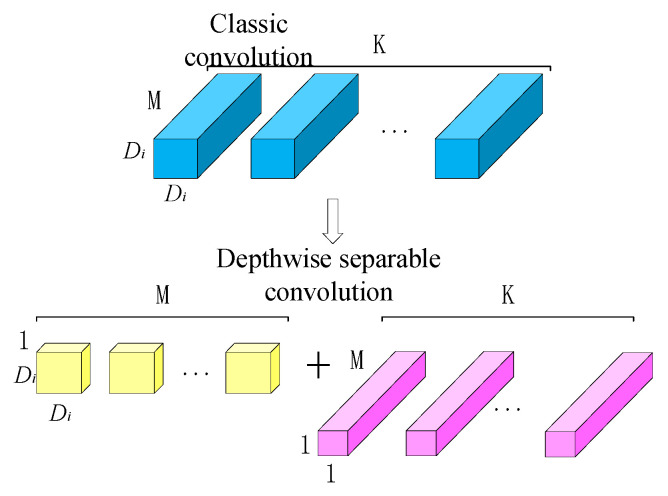
Classic convolution and depth separable convolution.

In depth separable convolution, DW and PW are performed separately, as shown in Figure 5. The calculation amount $C_{2}$ of the depth separable convolution can be calculated as Formula (2):

(2) $$C_{2}=D_{z}\times D_{z}\times M\times D_{i}\times D_{i}+K\times M\times D_{z}\times D_{z}$$

The calculation amounts of the depth separable convolution and classic convolution are compared as follows:

(3) $$\frac{C_{2}}{C_{1}}=\frac{D_{z}\times D_{z}\times M\times D_{i}\times D_{i}+K\times M\times D_{z}\times D_{z}}{D_{z}\times D_{z}\times M\times K\times D_{i}\times D_{i}}=\frac{1}{K}+\frac{1}{D_{i}^{2}}$$

In the equation, the channels number of the convolutional layer K is usually greater than 1, and the commonly used sizes of the convolution kernel are 3 × 3 and 5 × 5, so the result of the formula is less than 1. The calculation amount of the depth separable convolution is smaller than that of the standard convolution.

**Figure 6:**
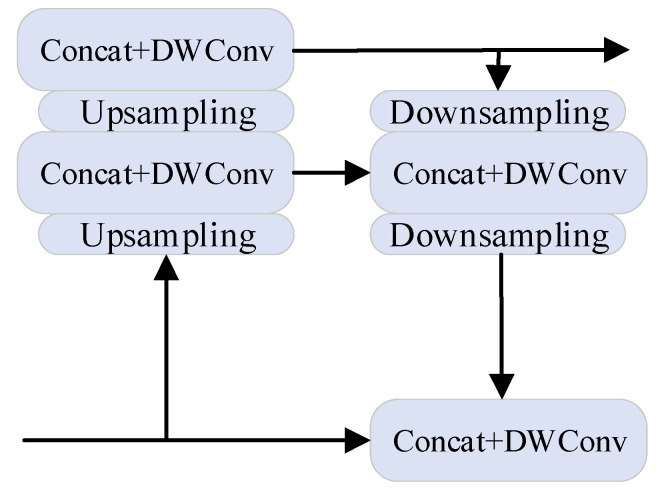
Improved PANet layer.

The PANet layer is improved, as shown in Figure 6. It can retain the advantages of PANet dynamic feature pooling, fully connected layer fusion and bottom-up path enhancement and also reduce the computation in PANet, so as to realize the lightweight of the network and, finally, achieve the optimization of YOLOv4.

## 4. Case Studies

### 4.1. Image Acquisition

**Figure 7:**
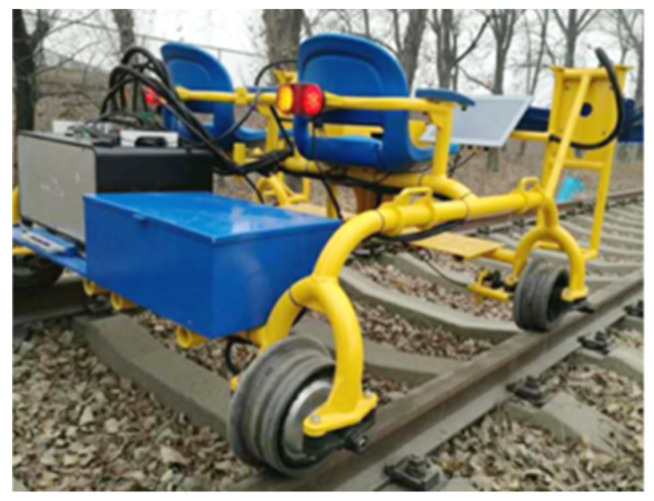
Intelligent track inspection vehicle.

According to the technical route of the proposed method, a track inspection field test was carried out in this paper. As shown in Figure 7, the intelligent track inspection vehicle used in the test was developed by Beijing Yinglu Technology Co., Ltd. (Beijing, China). The vehicle is composed of two parts: an electric inspection vehicle and a track state inspection system. The electric inspection vehicle contains a car body, track wheels and seats; the track state inspection system is composed of a host and a high-definition linear image scanning module. In this test, a 15 km track on the Beijing–Shanghai high-speed rail line is chosen as the test section. The travel speed of the inspection vehicle is 20 km/h, and the image resolution is 2048 × 2048.

The specific collection equipment data is shown in Table 1.

The specific configuration of the algorithm environment used in the test is shown in Table 2.

**Figure 8:**
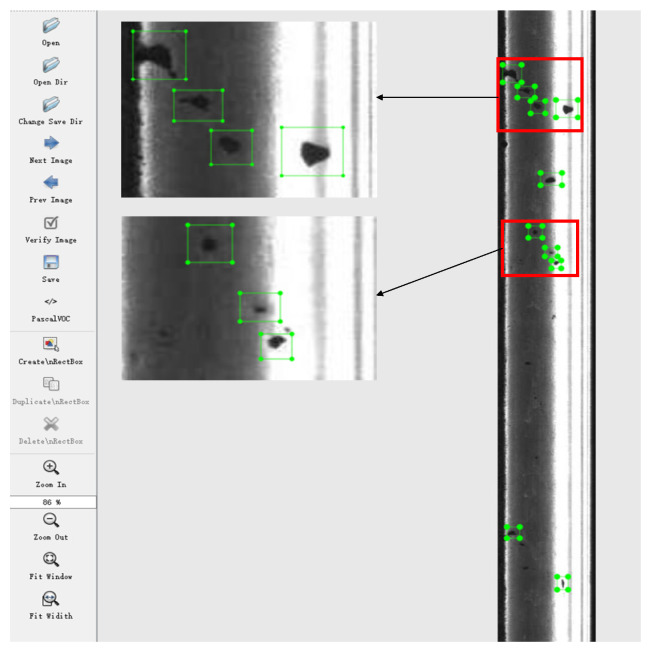
Image annotation.

One thousand rail images collected in the field test are chosen for rail surface defect detection; among which, 900 are randomly selected as the training dataset and 100 as the test dataset. Before applying the improved YOLOv4, it needs to operate image annotation to establish a dataset feature database. In this paper, LABELIMG software with version 1.0 was used for image annotation. LABELIMG is an image annotation tool that is written in python and uses QT as a graphical interface. The rail surface in the image is regarded as the target detection area, as shown in Figure 8.

After annotation, the coordinates of the rail surface defect area are obtained, and the training algorithm and the defect detection test are performed on the coordinate dataset generated by the image annotation.

### 4.2. Establish a Detection Model for Rail Surface Defects

**Figure 9:**
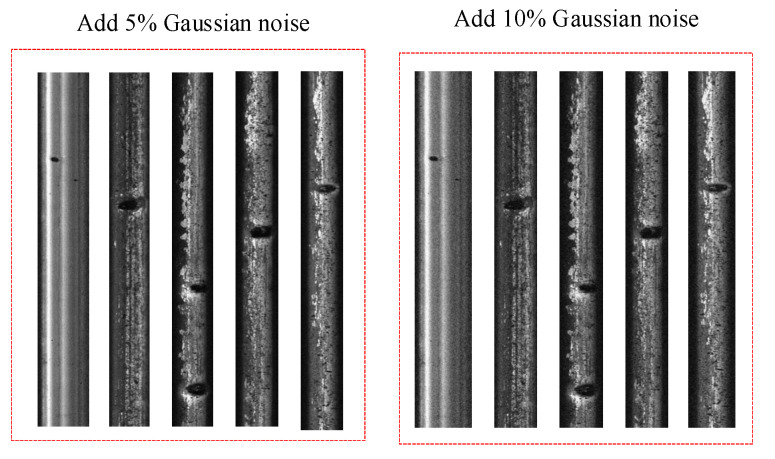
Gaussian noise processing diagram.

In order to verify the effectiveness of the method proposed in the study, 5% and 10% Gaussian noise are added to the original dataset, respectively, as shown in Figure 9.

**Figure 10:**
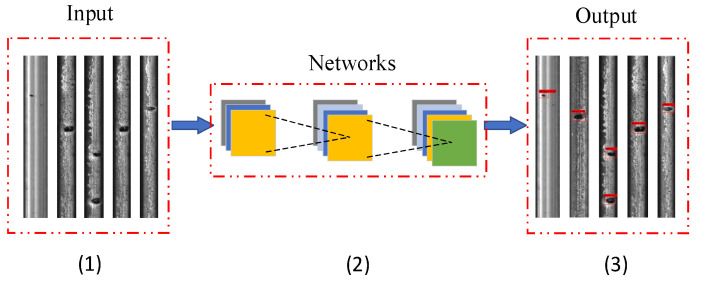
Rail defect detection model. (1) to reset the size of the input image; (2) to apply the improved YOLOv4 network based on the image operation; (3) to output the detection target.

The improved YOLOv4 uses MobileNetV3 as the backbone network of the feature extraction and, at the same time, uses deep separable convolution to replace the traditional convolution in PANet. A rail defect detection model is established, as shown in Figure 10, (1) to reset the size of the input image, (2) to apply the improved YOLOv4 network based on the image operation and (3) to output the detection target.

**Figure 11:**
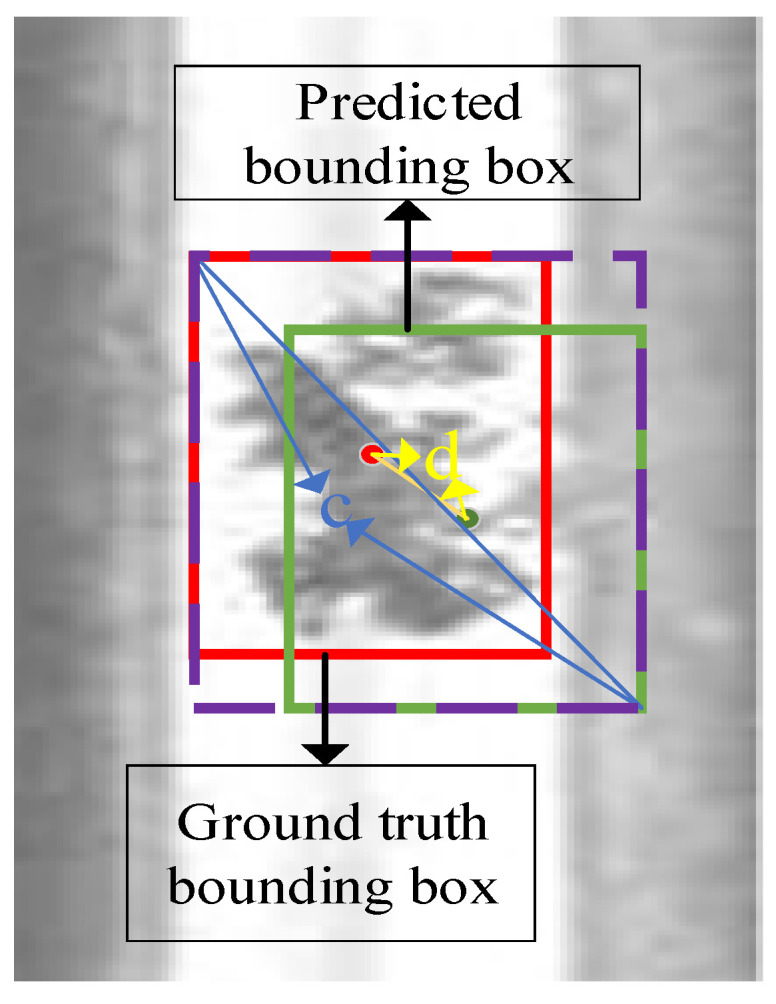
Comparison of the parameters of the methods.

The *CIOU* calculation method in YOLOv4 will make the target frame regression stable. It takes into account the distance, overlap, scale and penalty items between the target and the anchor point, and there will be no training divergence problem. Figure 11 illustrates the surface defects of the rail, and the red box indicates the target frame in which the rail surface defects are surrounded. The green box is the prediction box, and the purple box is the smallest rectangle that can cover the above two. d represents the center point distance between the target box and the predicated one. c represents the diagonal distance of the smallest area simultaneously covering the prediction box and the target box.

*CIOU* calculation formula is as shown in Formulas (4)–(6):

(4) $$v=\frac{4}{\pi^{2}}{\left(\arctan\frac{w^{gt}}{h^{gt}}-\arctan\frac{w}{h}\right)}^{2}$$

(5) $$\alpha =\frac{v}{1-IOU+v}$$

(6) $$\mathrm{CIOU}=\mathrm{IOU}-\frac{\rho^{2}(b,b^{gt})}{c^{2}}-\alpha v$$

where ρ refers to Euclidean distance; b, w and h refer to the center coordinates, width and height of the prediction box and $b^{gt}$, $w^{gt}$ and $h^{gt}$ refer to the center coordinates, width and height of the frame.

In the study, the *CIOU* threshold was set to 0.7. The detection image can be output only when the result is greater than 0.7, which makes the bounding box more accurate.

In the establishment of the rail defect detection model, the learning rate and the step size for each update is too large; thus, the model cannot converge on the extreme optimal value. If the learning rate is too small, the convergence can be guaranteed, but the efficiency of the model is sacrificed.

In order to avoid the above-mentioned problems, trade-offs have to be considered by modifying the model parameters with the best performances. The adaptive learning rate is used in the experiment to improve the optimization speed of the model, and the initial value of the learning rate is set to 0.001. In the training process, after each epoch, the current model loss and accuracy are evaluated in the training set, and the loss value change is detected every other epoch. When it is less than 0.0001, the learning rate *lr* is attenuated. The attenuation formula is expressed as Formula (7):

(7) $$lr^{*}=lr\times 0.1$$

### 4.3. Result Analysis

In the study, the same dataset is applied on the Faster R-CNN, YOLOv3 and YOLOv4 methods to compare and verify the effectiveness of the proposed method.

**Figure 12:**
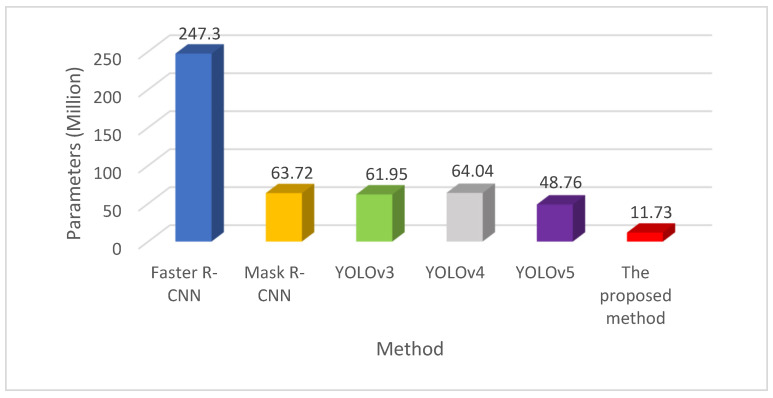
Comparison of the parameter quantities of the methods.

Figure 12 illustrates the comparison of the parameter quantity of each method. It shows that the parameter quantity in the proposed method is the least, which is about 1/20 of the Faster R-CNN. Since YOLOv4 is improved from the basis of YOLOv3, the parameter quantities of the two are not much different. Improved from the basis of YOLOv4, the proposed method replaces lightweight MobileNetv3 as the backbone network and uses deep separable convolution for PANet to further reduce the amounts of the parameters. From Table 3, the parameter quantity in the proposed method is decreased by 78.04% compared with YOLOv4, effectively reducing the amounts of the parameters.

In order to evaluate the detection results of rail defects, precision ($P_{r}$), recall ($R_{e}$), mean Average Precision (mAP), Frames Per Second (FPS) and volume are introduced. Among them, mAP is a common parameter for accuracy evaluations of different target detection models. Specifically, it is the mean of the average precision (*AP*) of each query. FPS refers to the number of frames transmitted per second, and volume refers to the size of the memory occupied by the model. The specific calculation formula is as follows:

(8) $$P_{r}=\frac{TP}{TP+FP}\times 100\%$$

(9) $$R_{e}=\frac{TP}{TP+FN}\times 100\%$$

(10) $$AP=\int_{0}^{l}p(r)dr$$

(11) $$mAP=\frac{1}{N}\sum AP_{i}$$

where True Positives (TP) and False Positives (FP) are the number of rail defects detected correctly or not, respectively. False Negatives (FN) is the number of rail defects detected incorrectly. N is the number of defects in all the rails.

From the detection results of rail surface defects by various methods in Table 3, it can be seen that, as a popular traditional method in the two-stage field, Faster R-CNN has a higher accuracy, recall and mAP than YOLOv3 but a lower detection speed. A slow, large model size not suitable for lightweight real-time detection, YOLOv3 has the advantage of a faster detection speed and smaller model size, but its accuracy, recall rate and mAP and Faster R-CNN methods are small; YOLOv4 in the accuracy, recall rate, mAP and FPS ahead of Faster R-CNN and YOLOv3, and its detection speed and model volume still have room for improvement. The research method in this paper was improved from the basis of YOLOv4. Due to the use of lightweight MobileNet V3 as the backbone network and deep separable convolution to improve the PANet, the model volume was 0.22 times that of YOLOv4, and the accuracy was improved by 1.64% compared to YOLOv4. Compared with YOLOv4, the recall rate and mAP were increased by 1.16% and 2.54%, respectively. At the same time, the detection speed of the research method exceeded YOLOv4 by 10.36 frames per second, which can better meet the requirement of rapidity.

Due to the complex environment of the rail, the algorithm is required to have a good anti-noise performance. In order to test the noise resistance of the research, Gaussian noise was added into the dataset. Table 4 and Table 5 are the detection results of rail defects with 5% and 10% Gaussian noise, respectively. It can be seen the proposed method has a higher mAP than the other methods and has more superior performance when noise exists. As the same models are used with slightly different test data, the FPS and volume of each method are consistent with those in Table 3. The results of Table 3, Table 4 and Table 5 show that the proposed method in this paper has good performance and can be applied to lightweight steel rail surface defect detection.

## 5. Conclusions

The rapid, accurate and intelligent detection of rail surface defects is of great significance for ensuring the safe operations of railway vehicles. According to the characteristics of rail surface defect detection, a one-stage detection model based on deep learning was constructed for the detection of rail surface defects. Through experimental verification and comparative analysis, the following conclusions were drawn:

(1) In order to reduce the weight of the rail surface defect detection network, the YOLOv4 algorithm was improved. The backbone network of YOLOv4 was optimized, and the PANet layer in YOLOv4 was lightened and improved. It reduced the algorithm parameters, increased the detection speed and reduced the model size.
(2) In order to solve the problem of small objects detection, the improved YOLOv4 method was used in rail surface defect detection. The test results verified the effectiveness of the method.
(3) Establish training and test datasets and adding Gaussian noise processing to the datasets let us conduct the detection case studies. The analysis results showed that, compared with the traditional detection method, the proposed method had a higher detection accuracy.

In addition to the above conclusions, with the rapid development of object detection methods, the ideas proposed in this paper can be extended to different deep learning networks. At the same time, in order to verify the effectiveness of the proposed method and to avoid introducing more variables, image preprocessing was not introduced in this paper. It can be inferred that the accuracy of the defect detection can be further improved if the image is effectively preprocessed. Finally, if sufficient railway surface defect images can be obtained to establish datasets, statistical tests can be performed to achieve a full statistical analysis of the proposed deep learning approaches.

## Figures and Tables

**Table 1:** Track inspection vehicle camera parameters.

Camera Model	TVI-LCM-01
Voltage input range	2-K linear array image acquisition module
power	20–30 V DC
Protection class	120 W
Working temperature	−20 °C to + 70 °C
storage temperature	−40 °C to + 85 °C

**Table 2:** Test environment.

Project	Environment
Development language	Python 3.9
Development framework	PyTorch1.2
CPU	Intel(R) i7-9700 CPU @ 3.00 GHz
GPU	NVIDIA GeForce RTX 2080 Ti
Running memory	16 GB
Hard disk size	1 TB

**Table 3:** Comparison of the detection results of rail defects.

Method	$P_{r}$	$R_{e}$	mAP	FPS (Hz)	Volume (MB)
Faster R-CNN	89.36%	79.07%	87.32%	12.26	521.8
Mask R-CNN	90.62%	81.36%	89.18%	5.60	245.4
YOLOv3	87.23%	77.27%	86.74%	28.40	234.2
YOLOv4	92.48%	81.40%	90.98%	34.28	244.1
YOLOv5	93.06%	82.08%	92.16%	37.32	185.6
The proposed method	94.24%	82.56%	93.21%	44.64	53.6

**Table 4:** Comparison of the detection results of rail defects with 5% Gaussian noise.

Method	$P_{r}$	$R_{e}$	mAP	FPS (Hz)	Volume (MB)
Faster R-CNN	82.61%	75.22%	85.08%	12.31	521.8
Mask R-CNN	88.26%	77.93%	86.92%	5.53	245.4
YOLOv3	80.02%	72.73%	83.10%	27.07	234.2
YOLOv4	90.48%	79.36%	87.23%	35.92	244.1
YOLOv5	91.62%	80.14%	90.08%	38.54	13.6
The proposed method	92.44%	80.27%	88.42%	42.78	53.6

**Table 5:** Comparison of the detection results of rail defects with 10% Gaussian noise.

Method	$P_{r}$	$R_{e}$	mAP	FPS (Hz)	Volume (MB)
Faster R-CNN	79.35%	71.52%	80.65%	11.80	521.8
Mask R-CNN	85.48%	72.30%	81.23%	5.47	245.4
YOLOv3	75.36%	68.18%	74.40%	28.33	234.2
YOLOv4	88.89%	72.73%	83.02%	32.35	244.1
YOLOv5	91.62%	80.14%	90.08%	36.00	13.6
The proposed method	89.92%	79.63%	84.28%	43.42	53.6

## Data Availability

The data used to support the findings of this study are available from the corresponding author upon request.
